# Sudden Death Due to Cardiac Tamponade Secondary to Myocardial Infarction Ventricular Wall Rupture

**DOI:** 10.7759/cureus.30288

**Published:** 2022-10-14

**Authors:** Amit Patil, Nawal Singh, S Vigneshwaran, Shreekant Bharti, Shreepriya Sudhakar

**Affiliations:** 1 Forensic Medicine & Toxicology, All India Institute of Medical Sciences, Patna, Patna, IND; 2 Pathology/Lab Medicine, All India Institute of Medical Sciences, Patna, Patna, IND

**Keywords:** acute myocardial infarction, forensic autopsy, ventricle rupture, heart tamponade, sudden cardiac death (scd)

## Abstract

Sudden deaths due to natural causes are commonly seen in forensic practice, most of which are attributed to cardiovascular diseases. Cardiac tamponade (CT) is one of the causes of sudden cardiac death, with a fatal outcome usually detected at autopsy. While both trauma and non-traumatic causes are responsible for CT, it is a known complication of acute myocardial infarction leading to cardiac rupture that involves the ventricular wall, septum, and papillary muscles. We report the case of a 50-year-old male who collapsed suddenly and was declared dead in the Trauma and Emergency Unit of the hospital before admission. Autopsy findings revealed 250 mL of blood and a 206 g blood clot in the pericardial cavity with a vertical tear on the posterolateral surface of the left ventricle with focal hemorrhagic myocardium consistent with acute myocardial infarction. The cause of death was CT as a result of myocardial rupture due to acute myocardial infarction. The gross and histopathological findings were diagnostic and revealed thrombosis of the left circumflex artery, transmural myocardial infarction, and ruptured myocardium of the left ventricle.

## Introduction

Cardiac tamponade (CT) is a clinical condition caused by increased intra-pericardial pressure due to the rapid accumulation of blood, pus, other fluids, or gas in the pericardial space. The increasing pericardial pressure exceeds central venous pressure, thereby limiting the return of venous blood to the heart. The condition is almost invariably fatal and can cause sudden death unless the pressure is relieved by removing the fluid either by surgical process or needle pericardiocentesis [1]. It is common in forensic practice to encounter naturally occurring sudden and premature deaths in which cardiovascular etiologies are more common. CT is one of the causes of sudden cardiac death reported in previous studies, and its prevalence is between 25% and 30% of large pericardial effusions [2-4]. As its presentation is sudden and rapid, with a fatal outcome, the diagnosis of CT is usually made at postmortem examination.

CT is more frequently associated with cases of trauma, intra-pericardial rupture of great vessels, operative procedures, and secondary to myocardial rupture. It is also associated with other conditions such as central venous catheterization, open heart surgery, malignancy, and dissecting aneurysms involving the aorta [5]. Ventricular rupture is a rare but fatal mechanical complication of acute myocardial infarction (AMI) and is responsible for as many as 15% of total early deaths in patients with AMI [6]. CT is a known complication of post-AMI rupture that involves ventricular wall, septal, or papillary muscle rupture. The most common site for ventricular rupture is the lateral wall at the mid-level [1,7-14].

We report the case of sudden death with no history of any traumatic event or any sign of injury to the chest; however, the autopsy revealed CT developed due to ventricular free wall myocardial rupture as an effect of AMI.

## Case presentation

A 50-year-old male police officer collapsed onto the floor while having dinner with his colleagues. He was declared dead on arrival at the hospital. Because the death was sudden and unexplained, a medicolegal autopsy was done to determine the manner and cause of death. According to the history given by the investigating officer (IO), the deceased was suffering from diabetes and hypertension but was irregular in his treatment. No further details regarding his medication and personal habits could be elicited. On examination, the deceased weighed 74 kg with a height of 174 cm. He was of average build and nourishment. Rigor mortis was well-developed throughout the body. Postmortem lividity was present on the back and dependent parts of the body, fixed in nature. There was no external injury to the body except for one reddish-colored contusion measuring 6 cm × 5 cm over the medial aspect of the left leg.

**Figure 1:**
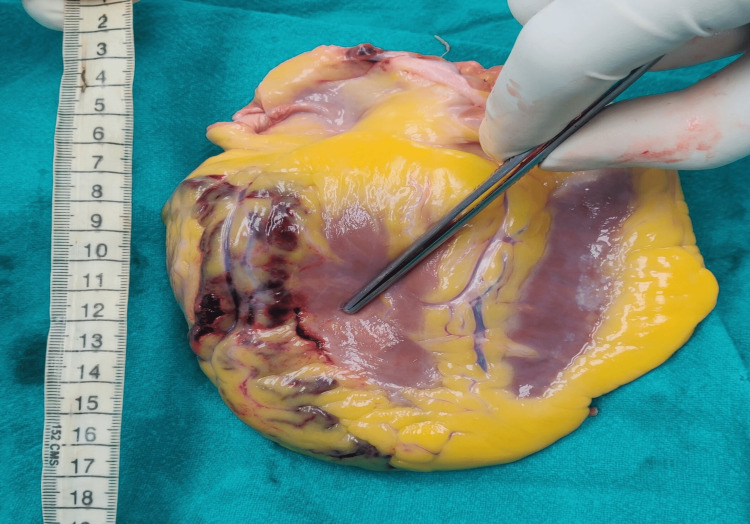
Vertical tear on the posterolateral surface of the left ventricle.

The thorax was opened after disarticulating both the sternoclavicular joint and opening the rib cage by cutting the ribs at the costochondral junction. The sternum was removed, beneath which the underlying pericardium was tense with purplish discoloration. The pericardial cavity contained 250 mL of blood and a 206 g blood clot in situ. The heart weighed 435 g conical in shape, with size being within normal limits in correlation to the extent of the victim. The other organs weighed the following: liver - 1,980 g, brain - 1,258 g, spleen - 250 g, right lung - 500 g, left lung - 625 g, right kidney - 250 g, and left kidney - 234 g. A vertical tear of 3 cm × 0.3 cm communicating with the ventricular cavity was seen on the posterolateral surface of the left ventricle and 2.5 cm proximal to the apex (Figure 1).

**Figure 2:**
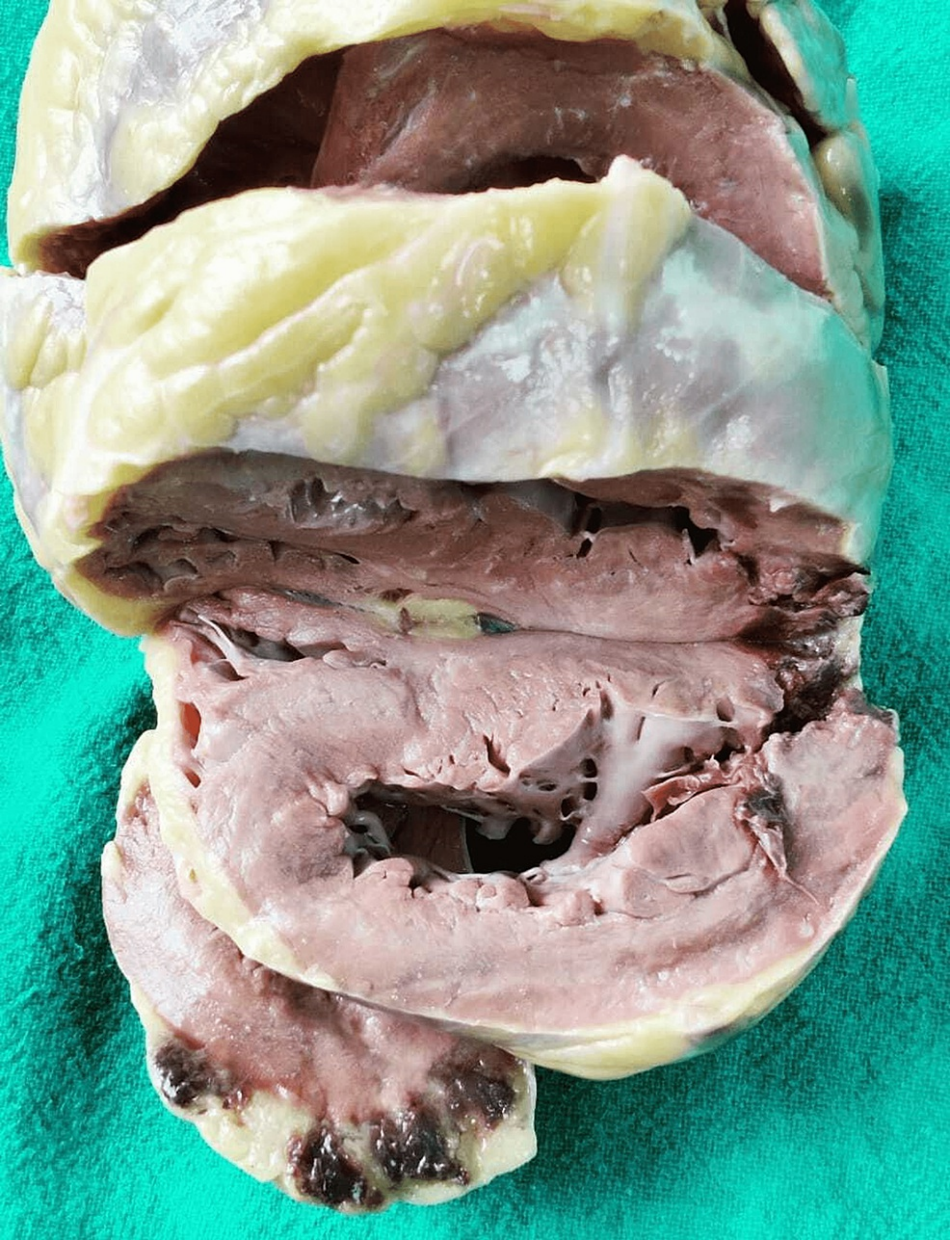
Acute myocardial infarction with ventricular free wall rupture and foci of hemorrhages in the epicardial fat.

A serial section of the left coronary artery showed subtotal atherosclerosis. In addition, the left anterior descending artery showed atherosclerotic stenosis with 40% narrowing of the lumen, 2 cm from its origin; the left circumflex artery showed 90% narrowing of the lumen, 1 cm from its source; the right coronary artery was patent. The serial transverse section of the heart revealed focal hemorrhagic areas suggestive of AMI associated with cardiac wall rupture of the left ventricular myocardium (Figure 2). Multiple hemorrhagic foci were present in epicardial fat on the right and left lateral walls. The rest of the heart structures, such as valves, cavities, and thickness of the ventricular walls, were normal. Generalized atherosclerosis was seen in the aorta (ascending, arch, and abdominal) and basilar arteries. All other internal organs were congested. The cause of death was CT as a result of myocardial rupture due to AMI. However, the heart was preserved for a gross and microscopic histopathology examination.

**Figure 3:**
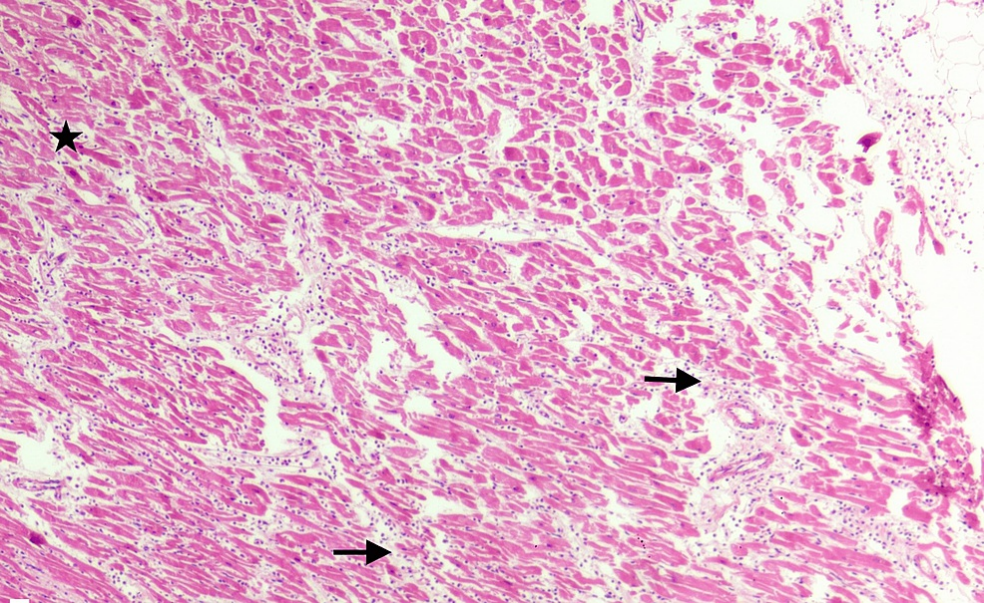
Acute myocardial infarction (hematoxylin and eosin stain, 100× magnification).

**Figure 4:**
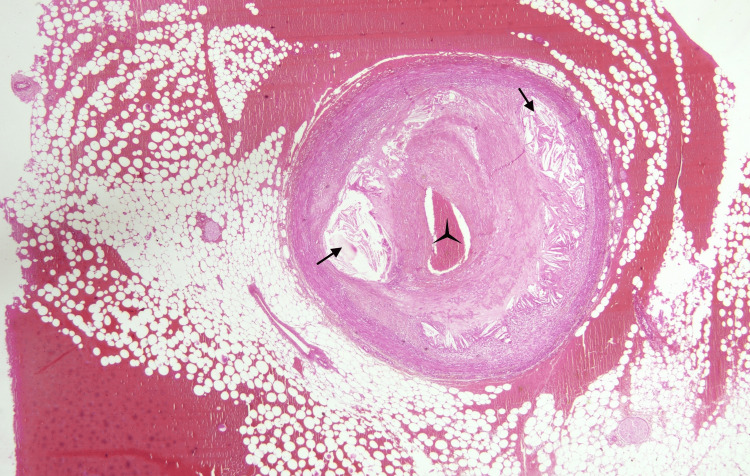
The left circumflex artery showing near-total occlusion with an intraluminal hemorrhagic thrombus (hematoxylin and eosin stain, 100× magnification).

**Figure 5:**
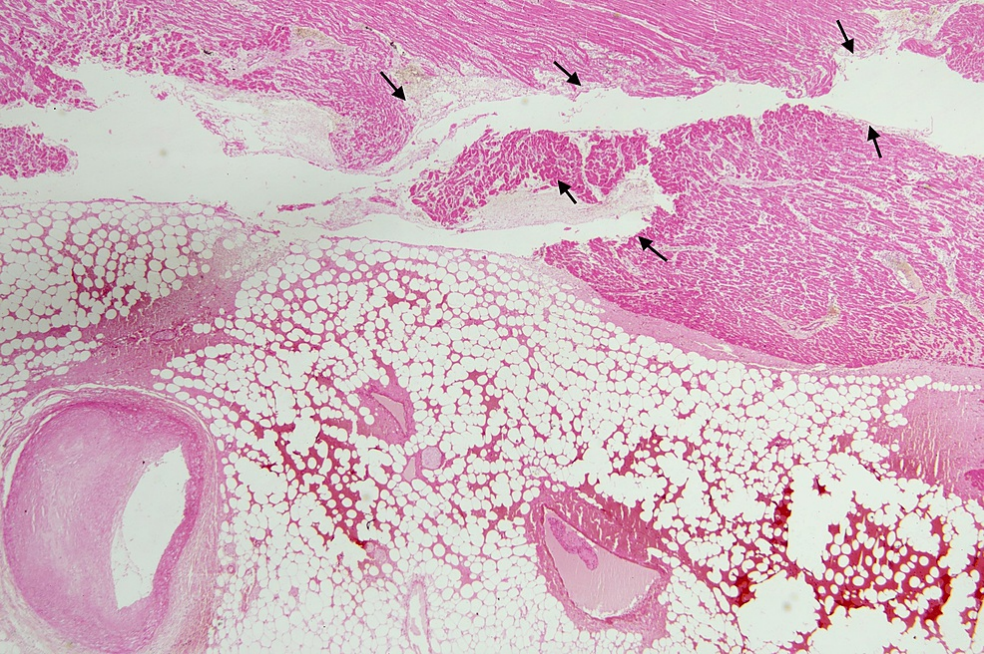
Acute myocardial rupture (hematoxylin and eosin stain, 100× magnification).

Further, the gross histopathology examination of the heart revealed significant atherosclerotic occlusion and thrombosis of the right coronary artery and left circumflex artery. The heart was sliced with a serial transverse section. The left myocardium showed focal pale discoloration suggesting recent and old myocardial infarction. A total of 32 tissue blocks were taken from the formalin-fixed heart for histopathological examination and were stained with hematoxylin and eosin. On histopathology, the left ventricular wall showed diffuse acute inflammatory infiltrates between the muscle fibers (black arrows) and uneven, ischemic red myocardial bundles (black star). The affected area represented around 4-12-hour-old AMI (Figure 3). The left circumflex artery showed near-total occlusion with an intraluminal hemorrhagic thrombus (black tri-star) and abundant cholesterol clefts within tunica media (black arrows) (Figure 4). The ventricular wall myocardium showed rupture and disruption (black arrows) reaching the epicardial fat, mild hemorrhages, and a partially occluded coronary branch by atheromatous plaque (Figure 5).

## Discussion

The pericardium, primarily composed of dense fibrous tissue, acts as a protective layer of the heart. In addition, pericardial fluid of about 50 mL in the cavity provides cushioning to cardiac activity. The pericardial cavity can accommodate the collection of any fluid accumulated slowly, but a rapid accumulation of fluid (200-300 mL) inside the cavity may cause a fatal outcome [8]. The accumulated fluid causes compression of the heart, affecting its movement, and leading to defective ventricular filling and reduced cardiac output. Consequently, coronary blood flow to the myocardium is also compromised due to rising intra-pericardial pressure, resulting in impaired myocardial function. Increasing intra-pericardial pressure also impedes venous return to the heart. In the absence of any immediate intervention, a considerable amount of fluid is accumulated in the pericardial cavity in a short time, and, at one point, the increased pressure reduces the systemic venous-right atrium pressure gradient (cardiac filling) to a point where cardiac output is no longer able to maintain coronary artery and systemic perfusion, resulting in cardiovascular collapse [15-17].

This case illustrates sudden death due to CT, a catastrophic complication of post-myocardial infarction ventricular wall rupture. The deceased was a male who had a history of diabetes and hypertension on irregular medications. He had AMI due to a subtotal block of the left circumflex coronary artery with a fresh hemorrhagic thrombus. The infarction was transmural with ventricular free wall rupture causing CT.

The etiology of CT includes active and passive pericardial effusion due to trauma, iatrogenic intervention, rupture of AMI, or intra-pericardial rupture of a dissecting ascending aortic aneurysm [14,18]. Post-AMI rupture includes papillary muscle rupture, ventricular free wall, or septal rupture [8,18], and risk factors for this include age >60 years, female gender, pre-existing hypertension, and left ventricular wall hypertrophy [14]. In this case, the deceased had all the risk factors for ventricular free wall rupture except age and gender.

CT usually presents to the emergency department as sudden death and creates a problem of manner and cause of death. In such cases, the findings become apparent at the autopsy. CT has varied etiology which includes traumatic and non-traumatic causes. A case series reported three cases that suffered CT secondary to post-myocardial infarction wall rupture similar to this case and recognized CT as an uncommon complication of myocardial infarction [5].

## Conclusions

In the present case, the death was sudden due to CT secondary to myocardial infarction, an acute and fatal phenomenon. The gross and histopathological findings were diagnostic revealing thrombosis of the left circumflex artery, AMI, and ruptured myocardium of the left ventricle. CT is a life-threatening condition and needs early diagnosis and intervention. It mainly presents as sudden death, raising a high index of suspicion about the cause and manner of death.
